# The effectiveness of a shared conference experience in improving undergraduate medical and nursing students’ attitudes towards inter-professional education in an Asian country: a before and after study

**DOI:** 10.1186/s12909-015-0509-9

**Published:** 2015-12-23

**Authors:** Amelia ZE Chua, Daryl YK Lo, Wilbert HH Ho, Yun Qing Koh, Daniel SY Lim, John KC Tam, Sok Ying Liaw, Gerald CH Koh

**Affiliations:** Yong Loo Lin School of Medicine, National University of Singapore, National University Health System, Singapore, Singapore; Alice Lee Center for Nursing Studies, Yong Loo Lin School of Medicine, National University of Singapore, National University Health System, Singapore, Singapore; Saw Swee Hock School of Public Health and Yong Loo Lin School of Medicine, National University of Singapore, National University Health System, Singapore, Singapore

## Abstract

**Background:**

In recent years, increasing emphasis has been placed on the importance of collaboration within multi-disciplinary healthcare teams, so as to facilitate holistic patient care and thus allow improved treatment outcomes. There is hence an urgent need to educate healthcare undergraduates early in their professional careers on the importance of and complexities involved in cooperating with counterparts from other allied healthcare professions. In conjunction with this, a milestone student-led conference for undergraduate students, the 9th Student Medical-Nursing Education Conference (SMEC), was organised in 2013 to provide a unique opportunity for shared learning among the entire cohort of undergraduate medical and nursing students in Singapore matriculating in that year.

**Methods:**

This study evaluated the effectiveness of the 9th SMEC 2013 as a shared conference experience in improving the attitudes of undergraduate medical and nursing students in Singapore towards inter-professional education (IPE). A 19-point Readiness for Inter-Professional Learning Scale (RIPLS) questionnaire comprising three subscales was administered to participants both before and after the conference. 352 responses were collected, giving a response rate of 75.1 %. Results were analysed using paired-samples t-tests with statistical significance set at *p* = 0.05.

**Results:**

Improvements in overall scores for both medical and nursing students were reported for all three RIPLS subscales. Examining the RIPLS items individually, significant improvement in scores for both medical and nursing students was obtained in all 19 items. Prior exposure to IPE activities was not a predictor of improvement in IPE attitudes.

**Conclusion:**

The authors propose that student-led jointly-organised conference experiences are effective in improving healthcare students’ attitudes towards IPE. This study provides valuable insights to facilitate the development of further IPE programs to allow for the rapid and effective promotion of cooperation and collaboration between students across various healthcare disciplines.

## Background

Traditionally, doctors have been trained to be self-reliant and independent, with the profession relying more on expertise, autonomy and responsibility rather than interdependence, deliberation and dialogue [1]. In recent years however, increasing focus has been placed on the importance of team-based care and collaboration between various healthcare professionals.

Critical to this shift is the advent of inter-professional education (IPE). IPE can be defined as an “educational process through which students and practitioners are provided with structured opportunities for 'shared learning'”[2], allowing healthcare students to understand the intricacies of working together with members of other healthcare professions. “Working together” involves “acknowledging that all participants bring equally valid knowledge and expertise from their professional and personal experiences”, and can result in novel methods of problem solving[1], improving the effectiveness of patient care in the process while also allowing for superior treatment outcomes.

The literature suggests that IPE at the level of undergraduate learning could translate to improved working relations and understanding between the different healthcare professions. It is recommended that IPE be introduced early in the commencement of undergraduate healthcare courses, as this may help amend negative attitudes and avoid the formation of stereotypical views[2–4].

Medical and nursing students in Singapore have in fact responded positively towards the concept of incorporating IPE into their professional education[3]. It was hence decided that the 9^th^ Student Medical Education Conference (SMEC), the only student-led healthcare-focused conference for undergraduate medical students in Singapore, would be expanded to encompass both medical and nursing disciplines. The resultant 9^th^ Student Medical-Nursing Education Conference 2013 (9^th^ SMEC 2013) was aptly accorded the theme of “Under One Roof”, providing a milestone joint IPE event for first-year undergraduate medical and nursing students.

In this paper, the authors present an assessment of the effectiveness of the 9^th^ SMEC 2013 in improving attitudes of conference participants towards IPE.

### Research population

The 9^th^ SMEC 2013 was held for first-year medical and nursing students at the very start of the 2013–2014 academic year in August. Notably, with the opening of Singapore’s second undergraduate medical school that same year, the conference was able to transcend institutional boundaries as well, ultimately reaching out to all matriculating undergraduate medical and nursing students across the nation. Participants encompassed undergraduate medical students from the Yong Loo Lin School of Medicine, National University of Singapore and the Lee Kong Chian School of Medicine, Nanyang Technological University, as well as undergraduate nursing students from the Alice Lee Center for Nursing Studies, Yong Loo Lin School of Medicine, National University of Singapore. With this, the programme of the conference was especially tailored to ensure that all participants were able to gain insight from qualified professionals and educators in both the medical and nursing fields. Table 1 provides details on the IPE-focused plenary session and workshops that comprised the bulk of the conference.

**Table 1:** Inter-Professional Education Events in the 9^th^ SMEC 2013

Event	Description	
Plenary Session	Distinguished members of the medical and nursing fraternities in Singapore were invited to share their insights with conference participants. The speakers included: • Vice Dean (Education), Yong Loo Lin School of Medicine, National University of Singapore • Co-Chairperson, Inter-Professional Education Steering Committee, National University of Singapore • Emeritus Consultant, Department of General Surgery, Tan Tock Seng Hospital • Vice President, 64^th^ Medical Society, Yong Loo Lin School of Medicine, National University of Singapore • Vice President, 6^th^ Nursing Subclub, Alice Lee Center for Nursing Studies, Yong Loo Lin School of Medicine, National University of Singapore (Of note, the Vice-Presidents of the Medical Society and Nursing Sub-Club presented a joint address, personifying the practice of building good inter-professional relationships even during undergraduate education.)	
Small-Group Workshops	Participants were given the opportunity to select the workshop that interested them the most out of a choice of 15. Each workshop was co-facilitated by at least one doctor and one nurse; with some facilitators inviting additional colleagues to share at the session. Facilitators were given the freedom to conduct each workshop in any preferred format, but with the guideline that focus should be on the teamwork and cooperation between various healthcare professionals in daily practice. Activities chosen by the facilitators ranged widely from video presentations to role-playing and group discussions. Many also chose to use examples from key events in the history of healthcare in Singapore, such as the 2003 epidemic of Severe Acute Respiratory Syndrome (SARS). It was encouraging that all facilitators displayed a great keenness to share their thoughts on IPE with the conference participants. The 15 workshop topics were as follows:	
	• Emergency Medicine • Family Medicine • General Surgery • Geriatrics • Infectious Diseases • Internal Medicine (2 Workshops) • Maternal Health	• Neuroscience • Oncology • Paediatrics • Psychiatry • Research • Simulation • Social Medicine

(Additional non-IPE events held during the conference included a symposium on “Surviving Medical and Nursing School”, as well as a scientific poster competition and a symposium on hospital residency for senior medical students.)

## Methods

### Data collection

The conference was assessed via the administration of the Readiness for Inter-Professional Learning Scale (RIPLS) to all conference participants. The RIPLS was originally formulated in 1999 by Parsell and Bligh[5] as a 19-item questionnaire consisting of 3 subscales (Teamwork and Collaboration; Professional Identity; and Roles and Responsibilities). As the first instrument designed to evaluate the “readiness” of healthcare students for shared activities, the RIPLS allows educators to quantify the impact of interventions on healthcare students[6, 7]. RIPLS has subsequently been proven to be a valid and useful tool for measuring student attitudes towards IPE in the undergraduate context[8]. For each item, participants were asked to provide their response using a Likert scale with 1 representing “Strongly Disagree” and 5 representing “Strongly Agree”. The questionnaire was administered twice to all participants –before and after the IPE components of the conference (prior to the plenary session and after the small-group workshops) to determine the effectiveness of the conference in improving students’ attitudes towards IPE. Participation in this study was voluntary, with consent taken after provision of a Participation Information Sheet containing details of the study. The study was approved by the National University of Singapore Institutional Review Board.

### Statistical analysis

Six of the 19 items in the RIPLS were negatively worded in the survey form; however for the sake of presentation, the scores recorded in this paper are such that a higher score is always indicative of a more positive attitude towards IPE. Cronbach alpha values were calculated to determine the internal consistency of the RIPLS instrument in our study population. Paired-samples t-tests were employed for each of the 19 items, as well as the 3 subscale scores and overall total score in order to evaluate changes in the conference participants’ attitudes towards IPE between before and after the conference. Chi-squared (*χ*^2^) tests were used to compare RIPLS scores between those who had prior exposure to IPE versus those who did not, so as to evaluate the effect of prior exposure to IPE on changes in RIPLS scores before and after the conference. Statistical significance was set at the conventional *p* < 0.05.

## Results

A total of 352 responses were collected out of a total possible 469. 81.6 % of medical students from the Yong Loo Lin School of Medicine, 68.5 % of medical students from the Lee Kong Chian School of Medicine and 61.2 % of nursing students from the Alice Lee Center for Nursing Studies responded, giving an overall response rate of 75.1 %. The demographics of the students who responded to the survey are illustrated in Table 2.

**Table 2:** Responders’ Demographics

	Total (*N* = 352)	Yong Loo Lin School of Medicine (YLLSoM) (*n* = 244)	Lee Kong Chian School of Medicine (LKCMedicine) (*n* = 37)	Alice Lee Centre for Nursing Studies (ALCNS) (*n* = 71)
Age, mean (standard deviation)	19.0 (0.8)	18.8 (0.7)	19.6 (0.8)	19.5 (1.1)
Gender, *n* (%)				
Male	134 (38.1 %)	100 (41.0 %)	26 (70.3 %)	8 (11.3 %)
Female	218 (61.9 %)	144 (59.0 %)	11 (29.7 %)	63 (88.7 %)
Ethnicity, n (%)				
Chinese	315 (89.5 %)	222 (91.0 %)	35 (94.6 %)	58 (81.7 %)
Malay	11 (3.1 %)	3 (1.2 %)	0	8 (11.3 %)
Indian	19 (5.4 %)	14 (5.7 %)	1 (2.7 %)	4 (5.6 %)
Others	7 (2.0 %)	5 (2.0 %)	1 (2.7 %)	1 (1.4 %)
Year of study, *n* (%)				
1^st^	347 (99.7 %)	242 (100.0 %)	37 (100.0 %)	68 (98.6 %)
2^nd^	1 (0.3 %)	0	0	1 (1.4 %)
First exposure to inter-professional education, *n* (%)				
Yes	313 (89.4 %)	216 (88.9 %)	31 (86.1 %)	66 (93.0)
No	37 (10.6 %)	27 (11.1 %)	5 (13.9 %)	5 (7.0 %)

^a^Numbers may not add up to total because of missing responses

The internal reliability of the pre and post-conference questionnaires was assessed separately. Cronbach’s α coefficients of 0.809 and 0.861 respectively were obtained, indicating a high internal consistency of the RIPLS questionnaire used.

The results obtained from the participants’ responses are shown in Table 3 with respondents stratified according to their course of study (medicine or nursing). Improvements in overall scores for both medical and nursing students were observed for all three RIPLS subscales. The scores for both medical and nursing students also improved significantly for all 19 individual RIPLS items. Prior exposure to IPE activities was not a predictor of improvement in IPE attitudes.

**Table 3:** Pre-conference (Before) and Post-conference (After) Scores of Readiness for Inter-Professional Learning Scale (RIPLS)

Subscale 1: Teamwork & Collaboration		Students								
		Medical (*n* = 281)			Nursing (*n* = 71)			Total (*N* = 352)		
		Mean (±SD)		*p*-value	Mean (±SD)		*p*-value	Mean (±SD)		*p*-value
		Before	After		Before	After		Before	After	
1	Learning with other students will help me become a more effective member of a health care team.	4.36 (0.49)	4.57 (0.57)	<0.001	4.17 (0.53)	4.49 (0.56)	<0.001	4.32 (0.51)	4.55 (0.57)	<0.001
2	For small group learning to work, students need to trust and respect each other.	4.53 (0.53)	4.65 (0.56)	0.001	4.35 (0.59)	4.58 (0.53)	0.001	4.49 (0.54)	4.64 (0.55)	<0.001
3	Team-working skills are essential for all health care students to learn.	4.60 (0.51)	4.67 (0.54)	0.061	4.42 (0.58)	4.63 (0.51)	0.001	4.56 (0.53)	4.66 (0.54)	0.002
4	Shared learning will help me to understand my own limitations.	4.37 (0.55)	4.56 (0.61)	<0.001	4.20 (0.55)	4.51 (0.53)	<0.001	4.34 (0.55)	4.55 (0.59)	<0.001
5	Patients would ultimately benefit if health care students worked together to solve patient problems.	4.60 (0.51)	4.73 (0.52)	0.001	4.44 (0.56)	4.67 (0.53)	0.001	4.57 (0.52)	4.72 (0.52)	<0.001
6	Shared learning with other health care students will increase my ability to understand clinical problems.	4.40 (0.53)	4.65 (0.55)	<0.001	4.27 (0.56)	4.60 (0.52)	<0.001	4.37 (0.53)	4.64 (0.54)	<0.001
7	Learning with health care students before qualification would improve relationships after qualification.	4.44 (0.58)	4.53 (0.60)	0.025	4.23 (0.51)	4.49 (0.50)	<0.001	4.39 (0.58)	4.53 (0.58)	<0.001
8	Communication skills should be learned with other health care students.	4.46 (0.60)	4.59 (0.56)	0.002	4.30 (0.57)	4.55 (0.53)	<0.001	4.43 (0.60)	4.58 (0.55)	<0.001
9	Shared learning will help me to think positively about other professionals.	4.20 (0.61)	4.58 (0.58)	<0.001	4.07 (0.49)	4.51 (0.53)	<0.001	4.18 (0.59)	4.56 (0.57)	<0.001
	Total *(Total Score: 45)*	39.95 (3.59)	41.52 (4.14)	<0.001	38.41 (4.05)	41.00 (3.88)	<0.001	39.64 (3.73)	41.42 (4.09)	<0.001
Subscale 2: Professional Identity		Students								
		Medical (*n* = 281)			Nursing (*n* = 71)			Total (*N* = 352)		
		Mean (±SD)		*p*-value	Mean (±SD)		*p*-value	Mean (±SD)		*p*-value
		Before	After		Before	After		Before	After	
10	Shared learning with other health care students will help me to communicate better with patients and other professionals	4.38 (0.57)	4.59 (0.55)	<0.001	4.25 (0.53)	4.51 (0.50)	0.001	4.35 (0.57)	4.58 (0.54)	<0.001
11	I would welcome the opportunity to work on small-group projects with other health care students	4.38 (0.56)	4.60 (0.60)	<0.001	4.20 (0.58)	4.51 (0.56)	<0.001	4.34 (0.57)	4.58 (0.59)	<0.001
12	Shared learning will help to clarify the nature of patient problems	4.27 (0.60)	4.59 (0.57)	<0.001	4.14 (0.55)	4.46 (0.53)	<0.001	4.25 (0.59)	4.56 (0.57)	<0.001
13	Shared learning before qualification will help me become a better team worker	4.36 (0.65)	4.60 (0.57)	<0.001	4.26 (0.56)	4.44 (0.67)	0.018	4.34 (0.64)	4.57 (0.60)	<0.001
14*	I don't want to waste my time learning with other health care students	4.57 (0.81)	4.72 (0.74)	0.001	4.21 (1.07)	4.58 (0.89)	0.003	4.50 (0.88)	4.69 (0.77)	<0.001
15*	It is not beneficial for undergraduate health care students to learn together	4.53 (0.77)	4.70 (0.70)	<0.001	4.21 (1.09)	4.66 (0.70)	0.001	4.47 (0.85)	4.69 (0.70)	<0.001
16*	Clinical problem-solving skills should only be learned with students from my own department	4.35 (0.94)	4.55 (0.79)	0.001	4.01 (1.13)	4.51 (0.84)	<0.001	4.28 (0.99)	4.54 (0.80)	<0.001
	Total *(Total Score: 35)*	30.85 (3.27)	32.34 (3.32)	<0.001	29.26 (4.04)	31.64 (3.29)	<0.001	30.53 (3.49)	32.20 (3.32)	<0.001
Subscale 3: Roles & Responsibility		Students								
		Medical (*n* = 281)			Nursing (*n* = 71)			Total (*N* = 352)		
		Mean (±SD)		*p*-value	Mean (±SD)		*p*-value	Mean (±SD)		*p*-value
		Before	After		Before	After		Before	After	
17*	The function of nurses and therapists is mainly to provide support for doctors.	3.67 (1.05)	4.09 (0.96)	<0.001	3.89 (1.04)	4.08 (0.84)	0.085	3.71 (1.05)	4.09 (0.94)	<0.001
18*	I'm not sure what my professional role will be.	3.56 (0.87)	3.92 (0.83)	<0.001	3.70 (0.92)	4.03 (0.96)	0.007	3.59 (0.88)	3.94 (0.86)	<0.001
19*	I have to acquire much more knowledge and skills than other health care students	3.52 (1.09)	3.65 (1.12)	0.033	3.40 (0.95)	3.51 (1.09)	0.356	3.50 (1.07)	3.63 (1.12)	0.020
	Total *(Total Score: 15)*	10.74 (2.10)	11.67 (2.09)	<0.001	10.97 (2.38)	11.63 (2.42)	0.015	10.79 (2.15)	11.66 (2.16)	<0.001
	Total *(Total Score: 95)*	81.54 (7.36)	85.51 (8.08)	<0.001	78.53 (8.98)	84.12 (7.76)	<0.001	80.95 (7.78)	85.23 (8.03)	<0.001

*Indicates negatively worded items that have been reverse-scored

Thirty-seven conference participants were found to have had previous exposure to IPE activities. Healthcare students who had undergone previous IPE experiences had a significantly higher baseline score (pre-conference) as compared to those without such experiences. However, there was no significant difference in the improvement in scores between those who had prior exposure to IPE and those who did not.

## Discussion

Results obtained for all 3 RIPLS subscales showed overall significant improvements in scores, indicating that the 9^th^ SMEC 2013 was effective in improving the attitudes of Singaporean healthcare students towards IPE. Notably, these improvements were obtained for both medical students as well as nursing students, implying that the 9th SMEC was not only able to improve attitudes towards IPE in both groups, but was also able to promote the importance of teamwork specifically between these two healthcare professions.

Looking individually at the 19 RIPLS items; with the exception of a minority of questions, the baseline scores for both medical and nursing students were already high prior to the conference and additionally showed statistically significant improvements post-conference. This overall positive result is extremely encouraging as it not only indicates that IPE activities are highly effective in improving students’ attitudes, but also suggests that Singaporean healthcare students display a high readiness to participate in and learn from such activities. This reflects the findings of studies on IPE activities in other countries [7].

With regards to question 3 of subscale 1 (see Table 3); the baseline scores for both medical and nursing students were high. However, while the cohort of medical students who participated showed improved attitudes towards IPE as a whole, it is noted that for this particular question, only the score for nursing students showed a significant improvement post-conference, whereas that for medical students showed only a slight increase that was not statistically significant. We hypothesize that this result is due to medical students’ perception of doctors as being preeminent members of the healthcare team who work independently, such that medical students accordingly have a tendency to view teamwork and collaboration with less importance as compared to other healthcare students. This appears to be a global phenomenon, with studies from New Zealand, the United Arab Emirates and Sweden reporting similar results [6, 9, 10]. Notably, the literature indicates that such perceptions extend past graduation into working life as well, with doctors valuing teamwork less as compared to other healthcare professionals such as nurses and pharmacists [8]. It is thus particularly important to correct this perception when attempting to improve medical students’ attitudes towards IPE. The authors hence suggest that additional studies are necessary to further elicit the detailed reasons for the prevalence among medical students of these specific perceptions as well as to identify the best ways of correcting such beliefs within the context of IPE.

Healthcare students who had undergone previous IPE experiences had a significantly higher baseline score (pre-conference) as compared to those without such experiences. However, there was no significant difference in improvement in RIPLS scores between those who had prior exposure to IPE compared to those who did not. This suggests that the 9^th^ SMEC 2013 was able to prove insightful by the same extent even to those who had prior exposures to IPE. It is hence worth considering whether participation in multiple IPE events would allow one to have a linear or even an exponential increase in the extent to which students are able to appreciate and work with members of other healthcare professions.

A notable aspect of this study is that almost the entire cohort of Singapore undergraduate medical and nursing students matriculating in 2013 was surveyed. With participants comprising the majority of first-year students from both undergraduate medical and nursing schools in Singapore, and coupled with the high response rate of 75.1 %, this study provides a comprehensive indication of both the attitudes of local Singaporean medical and nursing students towards IPE, as well as the potential effectiveness of IPE initiatives in the Singaporean context.

As the 9^th^ SMEC 2013 was one of the few healthcare conferences that are organised for students, by students, the results of this study suggest that student-run initiatives can be highly effective in improving attitudes towards IPE. Given that ultimately, healthcare students make up the target audience of these initiatives, it is sensible that such initiatives be organised by fellow students who are most equipped to identify the needs of their current generation; being the most able to organise the fulfilling, informative and enjoyable IPE experiences for their peers. This is supported by literature which has concluded that student-run initiatives should not just be considered to be nonessential electives, but as cherished events that will fuel the growth of IPE [11].

Perhaps another factor contributing to the effectiveness of the 9^th^ SMEC 2013 was the timing at which it was conducted; specifically being approximately one week into the beginning of university education for the undergraduate student participants. Holding IPE events at such an early stage of education makes it easier to avoid the phenomenon of students pigeonholing members of other healthcare professions, a phenomenon extremely common in post-graduate education should students not have previously participated in IPE [9]. Studies have in fact shown that holding IPE events in post-graduate education is much less effective, due to healthcare professionals having already developed their professional identity and hence holding more rigid views on inter-professional collaboration [5, 6, 12]. Within the period of undergraduate education itself, the literature in fact indicates that students who have just joined health profession courses are more receptive to IPE as compared to students who are nearing the end of their course [13–15].

It is acknowledged that there are limitations to the study. It is noted that this paper is unable to evaluate the long-term impact of the conference on participants’ IPE attitudes post-graduation. At the time of writing, it was not possible to elicit this as the cohort surveyed is yet to graduate for several more years. However, the authors suggest that given the long timeframe, any follow-up studies will be limited in significance, as the scope of further IPE and clinical exposures that will affect participants’ attitudes are likely to vary significantly among participants by the time of graduation. Hence it would very likely be inappropriate for any such studies to draw direct links between the effects of the conference in first year and IPE attitudes post-graduation. Nevertheless, the inability to extrapolate the results presented here to predict changes in the long-run does not preclude the fact that the conference was able to have a significant impact on participants. Rather, the authors suggest that the possibility of such impacts having an effect only in the short-term means that further IPE exposures throughout the course of undergraduate education may be necessary to supplement the shared conference experience described in this study. Additionally, the vast majority of conference participants were of the same age; hence it may not be appropriate to generalize our results to graduate medical and nursing students, or students from non-Asian countries. The use of open-ended questions in addition to multiple-choice questions in the questionnaire could possibly be useful in allowing for a more comprehensive approach when studying this aspect in the future [16, 17]. A further extension to this study could be to consider if a similar conference targeted at other combinations of healthcare professions such as dentistry, pharmacy and occupational therapy for example would prove to be as effective. The authors also suggest that while IPE is known to have an important impact on improving patient care, it would be interesting to further explore the ways in which an improvement in IPE attitudes affects healthcare professionals’ specific working practices, possibly allowing for more targeted interventions to be implemented.

## Conclusion

Our study found that participation in a student-led jointly-organised conference event was effective in improving medical and nursing students’ improve attitudes towards IPE. For students barely into the first year of healthcare education, such an IPE event is extremely important and vital to developing a readiness to work with other members of the healthcare profession, a very important quality to possess especially in light of today’s scientific advancements that herald interdependent healthcare cooperation. IPE is a relatively new pedagogical tool in medical and nursing education, thus further research should be undertaken to elicit more ways in which IPE can be incorporated into the curriculum. It is hoped that this paper will assist others in conveying the concepts of fostering teamwork, collaboration, an awareness of one’s roles and responsibilities as well as uniqueness of one’s discipline through their own IPE experiences.
